# A systematic review of community-to-facility neonatal referral completion rates in Africa and Asia

**DOI:** 10.1186/s12889-015-2330-0

**Published:** 2015-09-30

**Authors:** Naoko Kozuki, Tanya Guenther, Lara Vaz, Allisyn Moran, Sajid B. Soofi, Christine Nalwadda Kayemba, Stefan S. Peterson, Zulfiqar A. Bhutta, Sudhir Khanal, James M. Tielsch, Tanya Doherty, Duduzile Nsibande, Joy E. Lawn, Stephen Wall

**Affiliations:** Department of International Health, Johns Hopkins Bloomberg School of Public Health, 615 N. Wolfe St. W5019, Baltimore, MD 21205 USA; Save the Children, 2000 L Street NW, Suite 500, Washington, DC 20036 USA; Aga Khan University, Karachi, Pakistan; Makerere University College of Health Sciences School of Public Health, Kampala, Uganda; International Maternal and Child Health, Department of Women’s and Children’s Health, Uppsala University, Uppsala, Sweden; Karolinska Institutet, Department of Public Health Sciences, Stockholm, Sweden; Center of Excellence in Women and Child Health, Aga Khan University, Stadium Road, P.O. Box 3500, Karachi, 74800 Pakistan; Center for Global Child Health, Hospital for Sick Children, 686 Bay Street, Toronto, ON M5G A04 Canada; Morang Innovative Neonatal Intervention/John Snow Inc. Research and Training Institute, Kathmandu, Nepal; Department of Global Health, George Washington University Milken Institute School of Public Health, 950 New Hampshire Ave., NW, Suite 400, Washington, DC 20052 USA; Health Systems Research Unit, South African Medical Research Council, Francie van Zijl Drive, Parow, Cape Town South Africa; School of Public Health, University of the Western Cape, Bellville, Cape Town South Africa; Health Systems Research Unit, South African Medical Research Council, 491 Ridge Road, Durban, South Africa; Maternal Reproductive and Child Health (MARCH) Center, London School of Hygiene and Tropical Medicine, Keppel Street, London, WC1E 7HT UK; Research and Evidence Division, UK AID, 22 Whitehall, London, SW1A 2EG UK

**Keywords:** Neonatal, Referral, Careseeking, Community health worker

## Abstract

**Background:**

An estimated 2.8 million neonatal deaths occur annually worldwide. The vulnerability of newborns makes the timeliness of seeking and receiving care critical for neonatal survival and prevention of long-term sequelae. To better understand the role active referrals by community health workers play in neonatal careseeking, we synthesize data on referral completion rates for neonates with danger signs predictive of mortality or major morbidity in low- and middle-income countries.

**Methods:**

A systematic review was conducted in May 2014 of the following databases: Medline-PubMed, Embase, and WHO databases. We also searched grey literature. In addition, an investigator group was established to identify unpublished data on newborn referral and completion rates. Inquiries were made to the network of research groups supported by Save the Children’s Saving Newborn Lives project and other relevant research groups.

**Results:**

Three Sub-Saharan African and five South Asian studies reported data on community-to-facility referral completion rates. The studies varied on factors such as referral rates, the assessed danger signs, frequency of home visits in the neonatal period, and what was done to facilitate referrals. Neonatal referral completion rates ranged from 34 to 97 %, with the median rate of 74 %. Four studies reported data on the early neonatal period; early neonatal completion rates ranged from 46 to 97 %, with a median of 70 %. The definition of referral completion differed by studies, in aspects such as where the newborns were referred to and what was considered timely completion.

**Conclusions:**

Existing literature reports a wide range of neonatal referral completion rates in Sub-Saharan Africa and South Asia following active illness surveillance. Interpreting these referral completion rates is challenging due to the great variation in study design and context. Often, what qualifies as referral and/or referral completion is poorly defined, which makes it difficult to aggregate existing data to draw appropriate conclusions that can inform programs. Further research is necessary to continue highlighting ways for programs, governments, and policymakers to best aid families in low-resource settings in protecting their newborns from major health consequences.

**Electronic supplementary material:**

The online version of this article (doi:10.1186/s12889-015-2330-0) contains supplementary material, which is available to authorized users.

## Background

An estimated 2.8 million neonatal deaths occur annually worldwide [1], three-quarters in Africa and Asia [2]. These deaths are due largely to preventable causes, with neonatal infection accounting for 24 % of all neonatal deaths. The proportion of deaths attributable to neonatal infection is even greater in high mortality settings [3]. Newborns surviving infection may also experience long-term, neurocognitive impairment; for example, about 15 % of surviving newborns with meningitis develop mild or severe impairment, and while data are sparse, it is plausible that there may be similar levels of impairment among newborns surviving sepsis [4]. These newborns are most vulnerable closest to the time of birth, and the timeliness of seeking and receiving care could make a difference in neonatal survival and prevention of long-term sequelae.

In low-resource community-based settings, careseeking for neonatal illnesses can be triggered through two paths. One is through household self-identification of potentially life-threatening illness and subsequent self-referral. Identification of danger signs by families gives the opportunity for the timeliest response, especially in settings where the coverage of health worker home visits is low or facility-based postnatal care is not readily available. However, the accuracy of family-identified danger signs has been found to be low [5]. The second path is via community-based health workers detecting danger signs during home visits, and referring the newborn for care at an appropriately equipped facility. For this purpose, the WHO and UNICEF recommend community health workers (CHW) make a minimum of two home visits for home births, the first occurring within 24 h of birth and the second occurring on day 3 [6]. Several studies have also included home-based treatment by a trained community-based health worker at the time of danger sign detection, prior to referral. A systematic review reported families in low- and middle-income countries (LMIC) seeking care for a median of 59 % (range 10-100 %) of neonates who were ill or suspected to be ill [7].

**Fig. 1:**
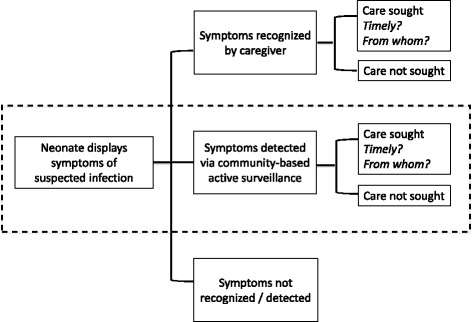
Components of neonatal careseeking. The shadded box is the focus of this paper. Figure adapted from Herbert et al. [7]

There is a gap in the literature in understanding how sick newborns are identified, treated, and referred, especially in programs that have CHWs conducting active surveillance for neonatal illness through home visits. There is also less information on neonatal referrals when compared to the more robust literature on infant or under-five referral, many arising from research on Integrated Management of Childhood Illness (IMCI). This paper focuses on the role active referrals by CHWs play in neonatal careseeking (Fig. 1). We examine referral completion rates for neonates with danger signs predictive of mortality or major morbidity in LMICs. We reviewed existing literature with information on referral completion rates following active home-based surveillance of neonatal illness, and also unpublished literature identified through a working group. We assess factors that may contribute to high or low completion rates, including infant sex, the danger signs assessed for referral, the overall referral rate, the ways in which referrals were facilitated, and other contextual issues.

## Methods

A systematic review was conducted in January 2012 and updated in May 2014 of the following databases: Medline-PubMed, Embase, and WHO databases (PAHO, IMEMR, WPRO, AFRO). We also searched grey literature using search engines on the following websites: Eldis, Healthy Newborn Network, UNICEF, UN, and USAID. The search consisted of phrases pertaining to developing country/list of all LMICs, neonatal, home-based, and referral; the full set of search terms for the Medline search is available in Additional file 1. No language or date restrictions were placed on the search.

In addition, an investigator group was established to identify unpublished data on newborn referral and completion rates. Inquiries were made to the network of research groups supported by Save the Children’s Saving Newborn Lives project and other relevant research groups. We sent a standardized data collection form to investigators and requested referral completion and vital status data (if available), stratified by the following characteristics: child sex, timing of referral (by week through 28th day), and level of facility the neonates were referred to. We also collected descriptive data on who made the referral, for what danger signs, whether anything was done to facilitate referral, and other pertinent information. The investigators of published data identified in the literature review were also contacted for additional information, as necessary.

We defined referral as newborns who were identified as ill or having one or more danger signs, and instructed to seek care from a facility by a community-based health worker, including trained volunteers, study staff, and those who are a part of the government health system. Referral completion was defined as having sought care from any qualified, facility-based provider. Whether the referral was completed was based on self-report and/or facility records. The study was included if all of the following conditions were met: (1) the study reported information on referral following health worker’s active surveillance during home visits; (2) the referral occurred in the neonatal period (within first 28 days of life); (3) the referral was for neonatal illness or danger signs; (4) a denominator of neonates assessed for danger signs was available and that there was minimal bias in the population assessed; (5) danger signs were assessed in a community-based setting by any community-based health worker or volunteer trained to detect danger signs; (6) those referred were systematically followed to determine completion of referral; and (7) the sample size of newborns referred was greater than 25. For any studies that also referred neonates for reasons other than illness (e.g. vaccination, general postnatal care), only data on illness referral were abstracted.

The studies that met the aforementioned criteria were summarized first into a matrix that captured the following data: study information (study name, geographic region, years of implementation, purpose of the study), description of the referral (research findings if the data came from a referral-related intervention study, frequency of household surveillance, whether home-based treatment was offered, whether/how referral was facilitated, what cadre of health workers referred the baby to where, the competencies and training of the health cadre, distance to the referral facility), availability of referral completion data (by early neonatal or neonatal period, by sex of child, time to referral completion, by severity of illness, self-referral rates, reasons/predictors of completion or non-completion), and other relevant information. Then the data were arranged into narrative form. Referral completion rates are also described in narrative form; because of the vast differences in context across the included studies, referral completion rates were not subjected to meta-analysis.

## Results

We identified 1318 publications in Medline and Embase, 160 on the WHO databases, and 5 in grey literature. From these, 58 publications were extracted for further review after examining titles and abstracts. Six studies were identified with relevant data [8–14]. The investigators of these studies were contacted for additional unpublished data, and responses were received from four research groups. One additional, unpublished source with relevant data (Aetiology of Neonatal Infections in South Asia study) and one doctoral thesis [15] which analyzed data from a randomized control trial in Nepal [16, 17] were identified (see Additional file 2: Figure S1 for search diagram). Table 1 has a summary of the included studies, and Additional file 3: Table S1 has further details. Included studies will hereafter be referred to by the country in which it was conducted and the year in which the study began.

**Table 1:** Description of included studies

Country and start year of study, official study name		Setting (years)	Purpose of study	Active surveillance	% of enrolled population who received home visit, at what timing	Referral facilitation	Referral from whom to where	Timing definition of referral completion
Asia	Bangladesh 2003	Rural Bangladesh (2003–2005)	RCT of home-based management of newborn infections by community health workers	Days 1, 3, 7, but if sick, daily visits made to complete antibiotic therapy and stress completion	Babies receiving at least one postnatal home visit increased from 46 to 79 % from beginning to end of study, timing unclear	Follow-up visit within 24 h for those who did not complete referral	Study staff (CHW) to government subdistrict hospitals, distinguished between completion to qualified vs. unqualified source, 38 % of those who sought care from qualified provider sought care from private sector	No timing indicated
	Projanhmo 1 [14]							
	Bangladesh 2004	Rural Bangladesh (2004–2006)	Study on improvement of household careseeking behavior through community health worker engagement	Days 1, 3, 6, 9, 28	73 % were assessed at least once, 54 % within first two days of birth	Referral slips, birth and neonatal care preparedness cards, referral tracking form, free care if coming to referral facility, system of emergency transport, training of TBAs	Study staff (CHW) to Kumudini Hospital, home-based care offered if refused	No timing indicated
	Projanhmo 2 [12, 13]							
	Nepal 2002	Rural Nepal (2002–2005)	RCTs of chlorhexidine application on newborn skin and/or umbilical cord for reduction of neonatal infections and associated mortality	Days 1, 2, 3, 4, 6, 8, 10, 12, 14, 21, 28	~62 % receiving first visit within 24 h, 96 % receiving first visit within 3 days	No facilitation	Study staff (CHW-level) to nearest facility	Care sought within 28 days of life
	Nepal Newborn Washing Study [15–17]							
	Nepal 2005	Rural Nepal (2005–2007)	Study on offering home-based care and referral for possible severe bacterial infection	Active (within 24 h of delivery), then passive up to 2 months. Graduation visit made at 2 months.	63 % within seven days of birth, 97 % for two-month follow-up visit	No facilitation	Female Community Health Volunteers to Facility Based – CHWs	Care sought within 28 days of life
	Morang Innovative Neonatal Intervention (MINI)^a^ [11]							
	Pakistan 2011	Rural Pakistan	Study on identification of etiologies of newborn sepsis	Days 2, 6, 13, 20, 27, 34, 41, 48, 59	90 % of enrolled newborns were followed at scheduled visits	Provided transport for referral to health facility. Follow-up visit within 24 h for those who did not complete referral	Study staff (CHW) to facility, study physicians at facility to tertiary facility	No timing indicated
	Aetiology of Neonatal Infection in South Asia (ANISA) (unpublished)^a^							
Africa	Ghana 2008	Rural Ghana (2008–2009)	RCT on improving neonatal health by training existing community-based surveillance volunteers to identify and conduct ANC/PNC visits	Days 1, 3, 7	63 % with at least one postnatal visit, 53 % in representative subsample had first visit on day of delivery or day after	Training and incentives for volunteers, materials and supervision for hospital newborn care strengthening and sensitization activities, referral card, counselled on keeping baby warm and frequent bf, dialoged and problem-solved around barriers, 24-h follow-up to check compliance, counselling at 2nd and 3rd PNC visits on five illness signs (stopped or poor feeding, too hot or too cold, difficult or fast breathing, jaundice, less active/lethargy)	Community-based surveillance volunteers to hospital or clinic, urban residents tended to comply to hospitals/clinics, rural residents to health center	No timing indicated
	Newhints^a^ [8, 34]							
	South Africa 2008	Urban South Africa (2008–2011)	RCT on home visit package to improve essential maternal/newborn care and PMTCT	First 24–48 h, day 3/4, day 10–14, within 3–4 weeks, after 6 weeks.	59 % received the first post-natal visit and of these 73 % within 48 h after discharge from hospital following delivery	Training and incentives referral slips, Partner Defined Quality approach to improve facility quality	Pre-existing CHW cadre to local PHC clinic	Care sought within 28 days of life
	Good Start^a^ [9]							
	Uganda 2009	Rural Uganda (2009–2011)	RCT on integrated maternal-newborn care package linking communities to facilities	Days 1, 3, 7, then quarterly surveys to follow up on referred newborns	N/A	Facility improvement, provision of referral forms, follow-up visit within 24 h	Pre-existing CHW cadre to hospital or health center grades II-IV (II and III are PHC clinics)	Within 24 h of referral
	Uganda Newborn Survival Study (UNEST) [10]^a^							

^a^Received unpublished data

### Study descriptions

Three Sub-Saharan African [8–10] and five South Asian studies [11–15] (one unpublished) reported data on community-to-facility referral completion rates. No datasets from the Americas were identified. The studies were conducted between 2002 and 2011. South Africa 2008 was conducted in a peri-urban setting, while the remaining were conducted in rural areas.

Six were randomized controlled trials, one was a non-randomized intervention study, and one was a study on identification of newborn sepsis etiology. Almost all included studies focused on neonatal danger sign detection, referral, and/or careseeking as a primary or secondary aim. Nepal 2002 [16], a randomized controlled trial on the application of chlorhexidine on the skin and/or umbilical cord for reduction of neonatal infections and mortality, was the only study that did not focus on those components. The timing and coverage of visits varied by study (Table 1). Three studies (Bangladesh 2003, Bangladesh 2004, and Nepal 2005) included a home treatment component for sick newborns. Two studies utilized pre-existing health volunteer cadres, two studies utilized pre-existing community health worker cadres, and four studies recruited and trained women in the community to conduct home visits.

Referral rates (or the percentage of newborns referred among those who received active surveillance) ranged widely, from 3 to 60 %. Danger signs that prompted referral were similar across most studies. The Young Infants Clinical Signs study established a seven-sign algorithm to detect illness in children under two months of age. The signs include difficulty feeding, convulsions, movement only when stimulated, respiratory rate of 60 breaths or more per minute, severe chest indrawing, fever (37.5° C or more), and hypothermia (below 35.5° C) [18]. While most studies assessed a large majority of these signs, if not all, for referral, several studies contained additional signs such as redness around the umbilicus, skin signs, jaundice, diarrhea, and birthweight cut-offs. The Nepal 2002 study had the highest referral rate of 60 % (the next highest rate being 21 % from Bangladesh 2004); 15 % were referred for severe skin infection (many pustules or blisters, OR one or more large areas of redness/pus), 15 % for cord infection Level I (moderate or severe redness that extended from the umbilicus to the skin OR moderate or severe swelling that extended from the umbilicus to the skin), 15 % for cord infection Level II (pus coming from the umbilicus AND redness AND swelling of any grade), and the remaining were comprised of various other signs. The primary aim of the Nepal 2002 trial, examining the impact of chlorhexidine application on the skin and umbilical cord for neonatal mortality reduction, most likely led to more careful and more inclusive detection of these particular conditions.

See Table 2 for the list of danger signs assessed in each study. Almost all of the studies had CHWs or community health volunteers referring from the home to a clinic or a hospital. Pakistan 2011 first had CHWs referring to study physicians, who then subsequently referred to facilities. Only one study referred to a non-clinician; the Nepal 2005 [11] study had Female Community Health Volunteers refer newborns to facility-based CHWs who did not have formal clinical training, but were trained to provide parenteral antibiotics to newborns. The competencies and the training of the CHWs were described in some studies; only half of the studies provided basic competencies and background of the CHWs and only half provided information on the training specific to neonatal danger sign detection (Additional file 3: Table S1).

**Table 2:** Neonatal danger signs surveilled in each study

Study	Feeding problem^a^	Lethargic/unconscious^a^	Fast breathing^a^	Severe chest indrawing^a^	Fever^a^	Hypothermia^a^	Convulsions/seizures^a^	Redness around umbilicus	Skin issues (pustules, abscess)	Weak cry	Jaundice	Vomiting	Diarrhea	Other
Bangladesh 2003	x	x	x	x	x	x	x	x	x	x	x	x		bulging fontanelle
Bangladesh 2004	x	x	x	x	x	x	x	x	x	x	x		diarrhea with blood in stool and/or dehydration	difficulty breathing 20 min after birth, eye pus
Nepal 2002		x	rapid breathing AND chest indrawing and/or fever		x	x		x	x		x		blood in stool, persistent loose/watery stools	
Nepal 2005	x	x	x	x	x	x		x	x	x				
Pakistan 2011	x	x	x	x	x	x	x							
Ghana 2008	x	x	x	x	x	x	x		x		x			very low birthweight
South Africa 2008	X	x	x	x	x	x	x	x	x			x		bulging fontanelle, baby stops breathing for a while but starts again after stimulation, swollen eyes, pus from baby’s ear, baby’s nostrils move outwards every time he/she breathes in
Uganda 2009^b^	x	very weak	x		x	x	x	x	x		x	x	x	excessive crying

^a^WHO Young Infants Clinical Signs

^b^Also referred for immunization and postnatal care, but those data were excluded from our analysis

The frequency of home visits during the neonatal period varied across the studies. Nepal 2005 [11], which was an operations research project, only requested one home visit within 24 h of birth by a Female Community Health Volunteer. After that, surveillance was conducted passively up to two months, or more specifically, families were instructed to notify the volunteer of any neonatal illness. In contrast, Nepal 2002 [16] had their CHW-level staff conduct 11 home visits in the neonatal period; this high frequency is attributable to surveillance for umbilical and skin infections associated with the study intervention (testing application of chlorhexidine on the skin and/or umbilical cord as an intervention for reducing neonatal mortality). The included studies planned a median of 3.5 visits during the neonatal period. Four studies conducted active surveillance only in the first week of life, of which one continued surveillance beyond that if illness had been detected. The scheduled timing of the first postnatal visit was within the first two days for all studies, with most studies scheduling a day 1 visit.

Four studies facilitated referrals, and the extent to which each study or program facilitated the referral varied widely. Ghana 2008 provided a referral card, which was reported as having elicited a sense of urgency from families. 73 % of the mothers perceived the cards as a confirmation that the baby is severely ill. Some families also reported that the card assured them the treatment would be sped up [8]. South Africa 2008 also provided referral slips, and improved quality of care at the referral facility via the Partnership Defined Quality approach [9]. Uganda 2009 [10] provided referral forms, had community health workers make a reminder visit within 24 h of referral, and also implemented referral facility improvement. Receiving the follow-up visit was predictive of higher completion. Bangladesh 2004 was the most extensive in intervening to increase completion; the study provided birth and neonatal care preparedness cards, training of traditional birth attendants, a referral slip for sick newborns, free inpatient care for those arriving with a referral slip, a system of emergency transport, and transport allowance to the poorest families [13]. Some studies offered home treatment prior to referral (Nepal 2005 [11]– oral cotrimoxazole, Bangladesh 2003 – intramuscular antibiotics [14]), while Bangladesh 2004 [13] offered home treatment (oral cotrimoxazole) if referral was refused or if the baby had at least one danger sign of very severe disease or at least two danger signs of possible very severe disease.

### Referral completion rate

Neonatal referral completion rates (n = 8 studies) ranged from 34 to 97 %, with the median rate of 74 %. The study reporting the lowest completion rate was Nepal 2002 [15]. In contrast, four studies reported completion rates above 90 % (Uganda 2009 [10], Nepal 2005 [11], Pakistan 2011, Ghana 2008 [8]). One study did not distinguish between self-referral (household seeking care of newborn without referral) and referral completion (Ghana 2008 [8]), and reported the two together as one care-seeking indicator (77 % of severely ill newborns sought care). One study (Bangladesh 2004 [13]) reported that while those seeking care at a facility designated by the study increased from 56 % to 80 % between the first month and third month of study implementation, the proportion of individuals arriving at the health facility with a referral slip decreased during the study period (72 to 41 %). This particular study’s intervention included behavior change communication messaging that sought to improve family recognition of danger signs, hence self-referral rates may have increased.

Four studies reported data stratified by whether referral was conducted in the early versus late neonatal period. Early neonatal (within first week of life) completion rates ranged from 46 to 97 %, with a median of 70 % (Table 3). Two of the studies reported statistically significantly higher completion rates in the late neonatal period (Table 4).

**Table 3:** Referral completion during the early neonatal period

Study	Sex	Referred (n)	Referral rate	Timing cut-off for completion	Referred and completed (n)*	Completion rate	Completion rate, w/o gender breakdown
Bangladesh 2004	No breakdown	554	Not available	Not indicated	272	0.49	0.49
Nepal 2002	male	4526	49.6 %	Within first 7 days of life	2390	0.53*	0.46
	female	4432	51.9 %		1734	0.39*	
Nepal 2005	male	155	4.3 %	Within first 7 days of life	142	0.92	0.90
	female	125	3.7 %		111	0.89	
Pakistan 2011	male	254	8.2 %	Within first 7 days of life	246	0.97	0.97
	female	247	8.7 %		242	0.98	

*The completion rates between male and female neonates were statistically significantly different (*p* < 0.05)

**Table 4:** Referral completion during the neonatal period

Study		Sex	Neonates referred (n)	Referral rate	Referred and completed (n)	Completion rate	Completion rate, w/o sex breakdown	Completion rate, stratified by early versus late neonatal periods	
								Early neonatal	Late neonatal
Asia	Bangladesh 2003	No breakdown	478	16.9 %	162 to qualified provider, 204 treated at home	0.34 complied to referral, 0.43 treated at home	0.34 complied to referral, 0.43 treated at home	N/A	N/A
	Bangladesh 2004	male	488	21.2 %	285	0.58	0.54	0.49**	0.61**
		female	431		210	0.49			
	Nepal 2002	male	7618	60.3 %	4145	0.54*	0.48	0.46***	0.51***
		female	7079	61.1 %	2894	0.41*			
	Nepal 2005	male	350	6.9 %	322	0.92	0.90	0.90	0.91
		female	322	6.7 %	286	0.89			
	Pakistan 2011	male	356	3.3 %	346	0.97	0.97	0.97	0.98
		female	310	3.1 %	303	0.98			
Africa	Ghana 2008	No breakdown	132	10.0 %	102	0.77	0.77	N/A	N/A
	South Africa 2008	male	30	6.1 %	28	0.93	0.93	N/A	N/A
		female	38		35	0.92			
	Uganda 2009^a^	No breakdown	327	Not available	243	0.74	0.74	N/A	N/A

^a^The study also referred for immunization and postnatal care, but those data were excluded

*The completion rates between male and female neonates were statistically significantly different (*p* < 0.05)

**The completion rates between the early neonatal period and the late neonatal period were statistically significantly different (*p* < 0.05)

***The completion rates between the early neonatal period and the late neonatal period were statistically significantly different (*p* < 0.05)

Only three studies reported time to completion. Uganda 2009 reported median completion of three days (IQR 1–6 days) [10], while South Africa 2008 [9] reported that only 24 % of mothers completed referral within one hour, 12 % delayed up to 12 h, and 63 % delayed for more than 12 h. Median completion was one day. The only determinant of early completion (defined as within 3 h) in this study was urban residence [8].

Five studies reported referral completion rates by infant sex, but only one had a statistically significant difference between boys and girls. Nepal 2002 [15] reported early neonatal referral completion rates of 53 % for boys and 39 % for girls and overall neonatal referral completion rates of 54 % for boys and 41 % for girls (both *p* < 0.001). The study reported that boys were more often taken for care, regardless of whether they were referred or of reason for referral. Also, the difference in careseeking was more notable when there were only female children prior to the child in question.

Bangladesh 2003 [14] defined referral completion as going to a qualified medical provider; the rates ranged from 34 % for very severe disease, to 25 % for possible very severe disease with multiple signs, and down to 10 % for possible very severe disease with a single sign. Bangladesh 2004 reported completion rates associated with each sign. Weak, abnormal, or absent cry (75.9 %), convulsion (74.8 %), and temperature above 101° F (73.2 %) reported the highest completion rates [12]. Those who had serious illness (defined as perinatal asphyxia, very severe disease, possible very severe disease, significant jaundice in the first day of life, possible gonococcal eye infection, diarrhea with blood, and diarrhea with severe dehydration) had over two times increased odds of completing referral (aOR 2.37, 95 % CI: 1.49-3.77). Nepal 2002 [15] stated that mothers of those with more severe danger signs (unconsciousness, fever, pneumonia, convulsions/stiffness, any two of difficulty breathing, vomiting, difficulty feeding, fever) tended to complete referral more. Uganda 2009 was the only study that referred for reasons other than illness (i.e. immunization). A newborn referred for illness had 2.3 higher odds of having a referral completed than a newborn referred for immunization (aOR 2.3, 95 % CI: 1.6-3.5) [10].

Two studies, Bangladesh 2003 and Bangladesh 2004, reported time trends in completion rate through the duration of their studies (two years). Bangladesh 2003 reported fluctuation in neonatal completion rate, shifting from 37 %, to 26 %, to 31 %, to 41 %, at each six-month cut-off from the beginning of their study. From the first six months to the last six months, care sought from an unqualified provider dropped from 28 % to 16 % [14]. Bangladesh 2004 reported an increase in completion from 56 % to 80 % between the first month and the third month of study implementation; the rate remained relatively constant thereafter [13].

Three studies reported reasons for and/or predictors of completion. In Bangladesh 2004, the most frequently raised reasons for completion included advice from CHWs that treatment is available at the referral facility (65.5 %), their understanding that the treatment was of high quality (34.5 %), and that treatment was free of charge (21.5 %) [13]. Ghana 2008 reported wealth quintile as the only statistically significant predictor; the highest quintile had a risk ratio (RR) of 0.77 (95 % CI: 0.66-0.90) for completion, meaning they complied less, compared to the poorest quintile [8]. However, it should be noted that the poorest completed the referral with less timeliness and to lower-level facilities. Uganda 2009 reported maternal age 25–29 and 30–34 respectively had decreased odds of completion compared to women age <20 (aOR 0.4, 95 % CI: 0.2-0.8 respectively) [10]. A reminder visit within 24 h of referral was a predictor of completion (aOR 1.7, 95 % CI: 1.2-2.7). It should be noted that these odds ratios include referral completion for immunization and postnatal care, in addition to referral completion for illness.

In three of the studies, non-completion could be differentiated by active refusal of care by a household versus non-completion due to barriers. In the Bangladesh 2003 study, 66 % refused referral, but of those, 65 % accepted home-based treatment from the same CHW who referred the newborn. The three most frequently listed reasons for non-completion were unavailability of someone to accompany the mother and the infant (24.7 %), child provided with traditional treatment instead (19.1 %), and bad weather or general strike (17.9 %) [13]. In Ghana 2008, the most frequently listed reasons for non-completion included the perception that the newborn was not severely ill (21 %), waiting for the infant’s health status to improve (18 %), and finances (18 %) [8]. South Africa 2008 had six refusals out of 110 referrals: two reported failing to recognize the severity of illness, two thinking no treatment was necessary, and two choosing other treatments [9]. One study offered home-based care if referral was refused. Bangladesh 2003 reported the lowest referral completion rates among the identified studies (34 %), but 43 % of families that refused care accepted home-based care [14]. Combined, close to 80 % of newborns received care for their illness.

Only three studies mentioned distance to facilities. The average distance to the referral government subdistrict hospital was 8.5 km for Bangladesh 2003 [14]. Ghana 2008 [8] reported that referral completion to the main referral facility was high within a 20 km distance, but the completion rate precipitously dropped beyond that distance. The South Africa 2008 [9] study, the only predominantly peri-urban/urban study included, reported that its study participants had no transport issues, as at least half of the mothers (56 %) could walk to the referral facility.

## Discussion

Existing data report a wide range of neonatal referral completion rates in Sub-Saharan Africa and South Asia following active illness surveillance. Several studies that we identified reported completion rates beyond 90 %, while one study reported a rate as low as 34 %. Interpreting these referral completion rates is difficult due to the great variation in studies; study design, programmatic approach and implementing context of each study, the danger signs criteria used for referral, whether and how referral was facilitated, and the frequency of surveillance visits are only few of the factors expected to influence the referral rate and referral completion rates.

The variation in completion rates may be superficial, due to differences in what percentage of newborns are referred. Taking Nepal 2002, this study was a nested pair of randomized controlled trials on chlorhexidine application to the skin and/or umbilical stump for reducing neonatal infections. The study referred around 60 % of the enrolled neonates, with a large majority being referred for skin and/or umbilical cord-related issues. Such a high referral rate would produce high sensitivity but extremely low positive predictive value for neonatal mortality or major morbidity. We would thus expect the lower referral completion rate (48 % for Nepal 2002), assuming families felt less urgency for less severe signs. In contrast, studies like Nepal 2005, Pakistan 2011, and South Africa 2008 reported referral rates in the single digits and completion rates in the 90th percentile. These studies have referral rates that are more comparable to expected rates of illness and bacterial infections, hence a higher positive predictive value for neonatal mortality or morbidity. If the studies with high referral rates reported completion rates just for severe danger signs, we may see completion rates as high as those studies with low referral rates. Also, this finding, along with available qualitative information, suggests that mothers’ perception of illness severity is a major consideration in improving completion rates. Darmstadt et al. reported for Bangladesh that a majority of mothers completed referrals for weak, abnormal, or absent cry (76 %), respiratory rate ≥70/min (75 %), temperature >101 F (73 %), all signs that are predictive of neonatal death. In contrast, hypothermia inspired lower completion rates (52 %), despite being similarly or even more predictive of neonatal death [12].

The included studies did not elaborate much on if and how referral facilitation impacted completion rates. In Bangladesh 2004, the mothers in the intervention group of the study received two pregnancy care and four postnatal care visits from community health workers, which led to increases in receiving treatment from qualified providers and referral completion rates [13]. Quality of referral messaging may also make a difference; studies have indicated that poor interpersonal communication skills of counselors correlated with referral completion rates [19]. In an IMCI study conducted in Ecuador, mothers who did not receive a referral slip and mothers who stated that the urgency of completion was not conveyed by the referent had lower completion rates [20].

Barriers to access, including cost, distance, and transport issues, often prevent mothers and/or families from seeking care. In Lucknow, India, an average episode of neonatal illness and hospitalization cost 547.5 and 4993 rupees (roughly $12 and $111 USD in 2006, when the study was conducted) respectively, when in the same community the mean monthly income was only 2804 rupees ($62 USD) [21]. In the studies included in our review, there was limited documentation on distance to and coverage of referral facilities, and inadequate stratified analysis in terms of how those factors may have impacted completion rates. Ghana 2008 was the only study in this review that examined distance as a predictor; shorter distance (<20 km) to the facility was a strong predictor of completion.

Several studies also showed that support to overcome such logistical barriers was not enough to inspire referral completion. Bangladesh 2004 offered free inpatient care and emergency transport, but only had a completion rate of 54 %. A neonatal infection study in Pakistan referred sick newborns from a primary care facility to a tertiary facility, and reported very low completion rates despite providing free transport and medical care [22]. The mothers cited other logistical barriers such as non-medical costs of hospital admissions (67 %) and lack of adequate facilities for those who accompanied the infant (45 %), but also cited reasons that led to the mothers or her family members actively rejecting care, like lack of permission from the father and/or elders (65 %) and religious and cultural beliefs (21 %). Additional studies have also noted similar reasons for active refusal of care such as fatalism regarding neonatal death or illness [23] (Uganda 2009, personal communication), traditional beliefs, (e.g. the need to confine mothers and newborns for certain number of days following birth) [24], and preference to receive treatment from traditional healers for all or certain danger signs [25–28]. Such complexities hint at the difficulty in designing interventions to remove all barriers to completing referrals.

The poor quality or perceived quality of care at referral facilities may also be a major barrier [29]. A study from India reported families’ preference to seek care from private facilities, even if they needed to borrow money, for they distrusted the quality of care at public facilities [30]. Bangladesh 2004 hypothesizes that improvement of care at the referral facility contributed to increased self-referral and referral completion. In addition to the actual quality of medical care in treating the illness, there are also concerns regarding the interaction between the facility staff and patients. Studies have reported perceived inappropriate behavior of doctors and nurses [31] and fear of judgment from nurses because they had delivered at home or were in difficult social or marital circumstances [23]. Other studies have reported prior unpleasant experiences at facilities as a reason for non-completion or refusal to seek care from a qualified facility [27, 29, 30]. There is also evidence of preferential treatment; one study reported that the strongest predictor of neonatal survival at the facility was whether the family knew a doctor at the facility [32], and a Pakistan study reported that families not speaking the dominant language was a predictor of low referral completion [22].

One of the main issues in synthesizing data on referral completion is the failure of studies and programs to define, document, and publish the nature of the referral and the referral completion. Only with proper documentation would we more objectively be able to assess what interventions would be effective in improving timeliness of careseeking, particularly for neonatal illness, and reducing neonatal mortality and morbidity. For instance, many of the studies included any careseeking up to 28 days as a completed referral or did not indicate the time span after which going to a facility no longer qualified as a completed referral. Only three included studies described time to referral completion, of which only two reported time to completion in units of hours instead of days. Because of the great vulnerability of newborns immediately after birth, the failure to define timely completion muddles our understanding of whether care was sought appropriately. Researchers and program implementers must better document time to completion and come to a better consensus on the timeframe an effective intervention should reduce the time to completion to. We recommend for future programs and studies to include documentation and publication of the following data: the breakdown of what signs/conditions newborns are being referred for, what was done to facilitate the referral, time between birth and the first visit of the CHW / referring individual (to assess whether these individuals are meeting the designated schedule for active surveillance), the background of the cadre of CHWs being used, time between referral and family mobilization for careseeking, time between this initial mobilization and receiving care, and the level of the facility the newborn was taken to (primary vs. tertiary), distance to the facility, and fees associated with care (Table 5). Furthermore, the self-referral rate as a complement to referral completion rate would be invaluable to document. Acknowledging that quantifying such data is difficult especially in programmatic settings, the distinction of time between referral and when the family mobilized to seek care and the time between mobilization and receiving care is invaluable. Such data will allow us to better target where exactly the largest delay is coming from in the careseeking timeline.

**Table 5:** Recommendations for documenting and publishing data on newborn referral completion

Referral characteristics	Referral for what danger signs
	Referral by whom
	● competencies of that health cadre
	● training received by that health cadre for danger sign detection
	● workload of that health cadre
	Referral to what level of care
	● primary care facility, tertiary facility, etc.
	● estimated distance or travel time
	● whether care is provided for free or with fees
	Was it facilitated and if so, how
	● include details (e.g. image of referral slips, content of behavioral change communication materials, etc.)
Quantification of referral completion	Time to first home visit of the referring individual following birth of child
	● time between family mobilization and receiving care
	Actual frequency and timing of home visits, in addition to the scheduled/expected frequency and timing
	Time between referral and family mobilization for careseeking
	Referral completion rate
	● definition of what qualifies as “complete” = defining the numerator and denominator of the rate clearly, e.g. including what the time cut-off is for seeking care following referral
	● stratified by any contextually important variables
	○ e.g. sex of child, socioeconomic and demographic background of family or mother (religion, ethnicity, education, etc.)
	Self-referral rate
	● definition
	● stratified by any contextually important variables

The interpretation of referral completion rates and neonatal careseeking is incomplete without taking into account self-referral. Self-referral is an important companion to referrals by health workers, as we expect limits in CHWs’ coverage and timeliness of home visits in programmatic settings [33]. Most of the included studies were research trials, and many included a large number of visits which is not likely to be feasible outside of a research setting [33]. Several of the included studies in our paper emphasized home-based identification of danger signs and subsequent careseeking, making self-referral rates as important an indicator of intervention impact as referral completion rates. However, very few of the included studies mentioned self-referral. Failing to account for self-referral may lead to underestimation of appropriate careseeking; if families are more likely to seek timely care on their own for what they perceive to be severe illness, those who are referred later may consist largely of mild cases, hence have lower referral completion rates. Another issue may be that the population that failed to self-refer may be a biased sample of those who are unable to seek care due to resource limitations. There is a need for more nuanced reporting of both referral completion and self-referral rates.

Completion rates are not static; data from the Bangladesh 2004 study is a good reflection of this point. In their study, they conducted extensive intervention to promote careseeking and reported completion rates of 56 % before implementation and 80 % after implementation [13]. Authors attribute this change to increasing awareness of signs of illness among family members and positive experience with services offered by the referral hospital; improving quality of care at the referral facility was a part of the intervention. While difficult to quantify, sharing of information through social networks could be contributing to such changes over time in completion rates.

As we noted, a large limitation of this study is the contextual differences of the included studies. Many factors influence a family’s decision to complete a referral for their newborn, making it difficult to arrive at a composition of interventions that would remove all barriers to timely referral completion. Furthermore, there was a dearth of available information in the literature, such as the details of the health cadre being used for referrals. To further understand this area, increased access to details of the tried interventions and tools may better guide future researchers and program implementers in elucidating what could best connect vulnerable newborns to appropriate care. Furthermore, the included studies did not include in-depth qualitative data on reasons behind completion or non-completion to accompany the quantitative data. Quantitative data alone fail to highlight how various mechanisms act on the family’s decision-making process pertaining to urgent neonatal care.

## Conclusion

600,000 neonatal deaths are attributable to neonatal infections annually. This burden does not include the undocumented number of surviving newborns who go on to experience long-term impairment from infections. Valid and early detection of newborn illness and subsequent timely careseeking from appropriate providers are critical in reducing this burden. Many factors contribute to the completion of referral and its timeliness. In order to both understand and differentiate these factors, studies and programs exploring this field must provide better documentation. Often, what qualifies as referral and/or referral completion is poorly defined in these studies, which makes it difficult to aggregate existing data to draw appropriate conclusions that can inform programming. Further research is necessary to continue highlighting ways for programs, governments, and policymakers to best aid families in low-resource settings in protecting their newborns from major health consequences.

## Additional files

Additional file 1:
**PRISMA Checklist.** (DOC 62 kb) Additional file 2: Figure S1. Literature search diagram. (DOCX 113 kb) Additional file 3: Table S1. Study description. (XLSX 48 kb)

## Abbreviations

CHW: Community health workers
LMIC: Low- and middle-income countries
IMCI: Integrated Management of Childhood Illness
